# Prevalence of Self-Medication among Students of Pharmacy and Medicine Colleges of a Public Sector University in Dammam City, Saudi Arabia

**DOI:** 10.3390/pharmacy5030051

**Published:** 2017-09-04

**Authors:** Fatimah Ali Albusalih, Atta Abbas Naqvi, Rizwan Ahmad, Niyaz Ahmad

**Affiliations:** 1 College of Clinical Pharmacy, Imam Abdulrahman Bin Faisal University (University of Dammam), Dammam 31441, Saudi Arabia; ph.fatimahali582@gmail.com; 2 Department of Pharmacy Practice, College of Clinical Pharmacy, Imam Abdulrahman Bin Faisal University (University of Dammam), Dammam 31441, Saudi Arabia; 3 Natural Products and Alternative Medicines, College of Clinical Pharmacy, Imam Abdulrahman Bin Faisal University (University of Dammam), Dammam 31441, Saudi Arabia; rizvistar_36@yahoo.com; 4 Department of Pharmaceutics, College of Clinical Pharmacy, Imam Abdulrahman Bin Faisal University (University of Dammam), Dammam 31441, Saudi Arabia; niyazpharma@gmail.com

**Keywords:** self-medication, pharmacy students, medical students, prevalence, epidemiology, Saudi Arabia

## Abstract

Pharmacy and medical students are expected to be more knowledgeable regarding rational use of medications as compared to the general public. A cross-sectional study was conducted among students of pharmacy and medicine colleges of Imam Abdulrahman Bin Faisal University in Dammam, Saudi Arabia using a survey questionnaire. The duration of the study was six months. The aim was to report self-medication prevalence of prescription and non-prescription drugs among pharmacy and medical students. The prevalence of self-medication in the pharmacy college was reported at 19.61%. Prevalence of self-medication at the medical college was documented at 49.3%. The prevalence of multivitamin use was reported at 30.53%, analgesics; 72.35%, antihistamines; 39.16%, and antibiotic use at 16.59%. The prevalence of anti-diarrheal medicines and antacids use among students was found to be 8.63% and 6.64%, respectively. The variable of college and study year was statistically associated with the nature of the medicines. The most common justifications given by students indulging in self-medication were ‘mild problems’ and ‘previous experience with medicines’. Our study reported that prevalence of self-medication in the College of Clinical Pharmacy was low, i.e., 19.61%. The figure has been reported for the first time. Students were mostly observed self-medicating with OTC drugs, however, some reported using corticosteroids and isotretenoin, which are quite dangerous if self-medicated. Students have a positive outlook towards pharmacists as drug information experts.

## 1. Introduction

Self-medication (SM) is a global phenomenon. It is prevalent in every age group, though its extent differs among individuals and regions. Previously, it was considered as unnecessary, however, responsible self-medication is regarded as an important aspect of self-care nowadays [1]. On the contrary, irresponsible or irrational SM is discouraged as it may not only harm the patient in the form of adverse drug reactions (ADRs) or medication-related problems (MRPs), but may also increase the direct costs, including the cost of treatment and hospital admission [2,3,4].

SM is defined as the use of medicines by a person for self-treatment based on self-diagnosed symptoms without consulting a physician and/or without a valid prescription [5]. It may incorporate over the counter (OTC) medications that are dispensed without prescription, as well as prescription-only medications (POM) which require a valid prescription, such as antibiotics. Though self-medication with POM is not advisable, the latter is common in those countries which do not have strict regulations on the sale of pharmaceuticals [6,7,8]. Self-medication practice offers ease of access to OTC medications at a lower cost, which serves as an alternative to the costly and time-consuming clinical consultations. Safety issues are a major concern as many diseases have similar symptoms. Additionally, the risk of self-medication is increased if the individual does not have knowledge and understanding of the disease. Additionally, this practice is associated with an increased risk of misdiagnosis, ADRs, drug abuse and misuse [9].

One of the reasons to indulge in this practice is the financial condition of the patient. This is common in those countries where the individual has to pay direct cost for treating the condition. As a result, patients may prefer self-treatment over costly consultation. Another possible reason can be the non-regulated practices concerning sale of prescription drugs. This may result in the availability of POM without a valid prescription and, hence, patient may skip consultation and directly purchase prescription medications [10]. Evidence indicates that self-medication is practiced by teenagers, adults, parents, and students in Saudi Arabia [11]. Familial practice may also render individuals to indulge in self-medication as it lowers their stigma towards SM. This highlights the need to educate parents about the problem as well. A pharmacist or a doctor at the community level can play an important role in this situation. This brings the discussion to the point of evaluating Saudi pharmacy and medical student’s outlooks toward this issue.

Pharmacy and medical students are expected to be more knowledgeable regarding rational use of medications as compared to general public. The curriculum of pharmacy and medicine teaches them about rational use of medicines and consequences of irrational use. Hence, this population is well aware of the phenomenon and issues related to this practice. Additionally, students of medicine would assume portfolio of a prescriber in future and may prescribe medicines. Similarly, pharmacy students would become future pharmacists and may find themselves counseling patients on safe use of medicines. Thus, both of these professionals play a significant role in patient care especially regarding this practice. Hence, understanding the practice and self-beliefs related to self-medication in this population is of paramount importance.

However, previous studies have reported that this population is also affected by the same practice [12,13]. A number of studies have been conducted in Saudi academia pertaining to the matter that reported a varying prevalence of SM. However, there were some limitations observed as those studies did not investigate the determinants, such as beliefs of students who either indulge or refrain from practice. Data regarding self-medication has not been reported from this academia before. Our study aimed to document this practice in pharmacy and medicine students at the university. It also had the objective of reporting prevalence of both POM and OTC medicines currently used by students at the campus.

## 2. Methods

A cross-sectional study was conducted among students of pharmacy and medicine colleges of Imam Abdulrahman Bin Faisal University (formerly known as University of Dammam) located in the city of Dammam in Eastern Province of Saudi Arabia. It was designed in the form of survey. The duration of study was four months, i.e., December 2016–April 2017.

### 2.1. Participants and Eligibility Criteria

The study included students of pharmacy and medicine colleges. This segment was identified as the target population of the study. Students of other colleges were not included. Additionally, students who did not consent to participate and incomplete questionnaires were also excluded from the study.

### 2.2. Sample Size and Sampling Procedure

The study employed purposive sampling to gather responses from students. The questionnaire was distributed among consenting participants. Students were approached in their free time (10 min) between their lectures and at prayer/lunch break. Additionally, students were allowed to take questionnaires to their homes and return them to the investigator at their time of convenience. The sample size was calculated online (Raosoft Inc., Seattle, WA, USA) [14]. According to official figures, the number of students enrolled in pharmacy and medical institutions in the country were 6242 and 15,559, respectively [15]. The sum of both figures was assumed as the total population, the confidence level was set at 95%, and the alpha margin of error kept at 5%. The sample size was found to be 378. The study was completed after gathering a total response from 450 students.

### 2.3. Research Instrument

A questionnaire was designed by reviewing the available research literature. It was designed in the English language, since it was the language of instruction at university colleges. Some of the research questions were incorporated from the research instrument developed by Naqvi et al. with permission [13]. The questionnaire was divided into two sections and consisted of 16 close-ended and four open-ended questions. The categories of variables identified were demographics of students that included age, gender, college affiliation, year of study, residence status, marital status, number of children, siblings, and past illness. All of these were included in Section 1 of questionnaire. Section 2 was concerned with self-medication information and included questions related to students’ beliefs about self-medication practice, indulgence in the practice, frequency of self-medication per month, source of information regarding the same, type and nature of medicines used, and place of obtaining medications, as well as common symptoms experienced prompting self-medication.

### 2.4. Piloting and Validation Process

The questionnaire was first presented to a panel of experts including college professors, health practitioners, and pharmacists. Based on their recommendations, the demographic variable of residence status was added in Section 1 of the questionnaire. Further to this, the panel recommended documenting the prevalence of self-medication in this population. For this purpose, two separate items related to indulgence in SM and frequency of SM per month, were added in Section 2. The questionnaire was then piloted in 180 students in pharmacy and medical colleges all over the Saudi Arabia to check for any errors. Some questions had spelling and grammatical errors which were rectified. It was observed that the categories for demographic variable of ‘number of children’ lacked the option of ‘not applicable’ for un-married respondents. This prompted confusion among respondents who selected the option ‘single’ in the marital information. This was identified and rectified by adding the required option. Furthermore, the two categories (df) of the demographic variable of ‘number of children’, i.e., ‘Between 3 and 5’ and ‘More than 5’ were merged together to increase data reliability.

The questionnaire was also statistically validated by employing reliability testing that revealed a Chronbach’s alpha value of 0.781. The exploratory factor analysis (EFA) using principle component analysis extraction and direct oblimin rotation with Kaiser Normalization was also employed, which extracted five components. The Kaiser Mayer Olkin (KMO) measure of sampling adequacy and Bartlett’s test for sphericity was also employed, which reported a value of 0.6 and a significant *p* value less than 0.001, respectively. Based on the statistical data, the questionnaire was distributed into five sections, namely: demographic information; prevalence information; common illness prompting SM; commonly used medications for self-use; reasons for/against SM and advice to others regarding SM practice. At this point, the questionnaire was deemed validated. The correlation matrix is presented in Table 1 and pattern matrix in Table 2.

**Figure 1:**
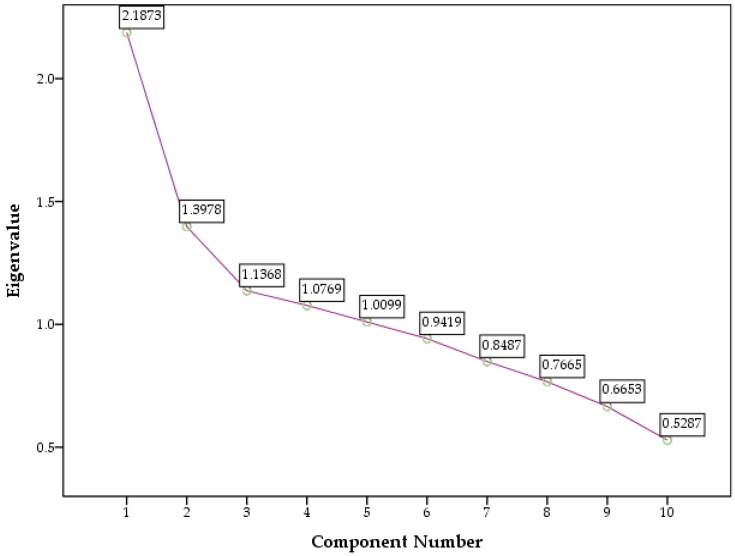
Scree plot.

The components with eigenvalues above 1.0 are graphically represented in Figure 1.

### 2.5. Data Coding and Analysis

The responses were analyzed using version 22 of SPSS (Statistical Package for Social Sciences) (IBM Corp., Armonk, NY, USA). The data was analyzed for frequency counts, and cross-tabulation was performed on associations. The chi square (X^2^) test was used to identify and report statistically significant associations. Prevalence was calculated by Medcalc^®^ and reported in terms of 95% confidence interval values [16,17]. An alpha margin of error (α) was identified at 0.05.

### 2.6. Ethical Approval and Participant Consent

The study was submitted for ethical approval to the ethics committee of the university. It was granted exemption from review (ID #2130001853). A verbal consent was sought from the respondents before handing the questionnaire. The participation was voluntary and without any incentive or pressure from investigators.

## 3. Results

### 3.1. Response Rate

The study incorporated students from pharmacy and medicine colleges. Out of N = 478 students, a total of 450 responses were received, giving a cumulative response rate (RR) of 94.12%. In terms of a college-wise response, a response rate of 96.8% was obtained from College of Clinical Pharmacy as 158 survey questionnaires were handed over to the pharmacy students and 153 were received. Similarly, a total of 320 surveys were handed to the medical students and 297 were received, giving a response rate of 92.81% for the College of Medicine. 

### 3.2. Demographic Information

The majority of students were aged between 18 and 23 years (N = 427, 94.9%). In terms of gender, slightly more than half were males (N = 249, 55.3%). Most of the students (N = 297, 66%) were from College of Medicine. Study year-wise breakdown revealed that most students (N = 138, 30.7%) were from 3rd Professional year, followed by 5th Professional year students (N = 103, 22.9%). The majority of students (N = 385, 85.6%) were single and did not have children (N = 385, 85.6%). A small segment of students were married without children (N = 43, 9.6%). Furthermore, the bulk of students (N = 375, 83.3%) lived with their families. Most of them (N = 195, 43%) had 3–5 siblings, followed by slightly more than a quarter of students (N = 124, 27.6%) who had 6–8 siblings. Amongst them, an overwhelming majority of students (N = 400, 88.9%) considered themselves healthy, while some (N = 10, 2.2%) had glucose 6–phosphate dehydrogenase (G6PD) deficiency and asthma (N = 8, 1.8%). The demographic information is tabulated in Table 3.

### 3.3. Self-Medication Information

The respondents were asked if they indulge in self-medication. More than half of students (N = 248, 55.1%) responded positively. The majority of students (N = 290, 64.4%) rarely indulged (once a month) in the practice, followed by a small segment (N = 54, 12%) that frequently (once every two weeks) indulged in SM. The overall prevalence of self-medication in the whole sample, i.e., among pharmacy and medicine students combined, was 26% (22.01–30.31% for a 95% confidence interval). The prevalence of self-medication among pharmacy students alone was reported at 19.61% (13.64–26.79% for 95% CI) and was 49.3% (44.84–53.77% for 95% CI) for medical students.

Most of the students (N = 124, 27.6%) obtained information from pharmacists followed by a quarter segment (N = 116, 25.8%) using the internet as well as obtaining information from physicians (N = 106, 23.6%). Almost half of students (N = 216, 48%) used non-prescription drugs, followed by a third (N = 152, 33.8%) using both OTC and POMs. An overwhelming majority obtained medications for self-use from a pharmacy store (N = 306, 68%), followed by a third proportion indicating availability of medicines in homes (N = 139, 30.9%). The most common symptoms experienced by students that prompted SM were headache, fever, pain, and dysmenorrhea (N = 64, 14.2%), followed by allergy and cold/flu (N = 16, 3.6%). The majority of students (N = 293, 65.1%) highlighted more than one symptom and few (N = 57, 12.7%) did not experience any symptom that prompted them to self-medicate. Further to this, majority of the students (N = 102, 22.7%) self-medicated with analgesics and antipyretics (paracetamol and NSAIDs), followed by cold and flu medicines (N = 10, 2.2%). A small segment (N = 6, 1.3%) self-medicated with antibiotics. 

**Figure 2:**
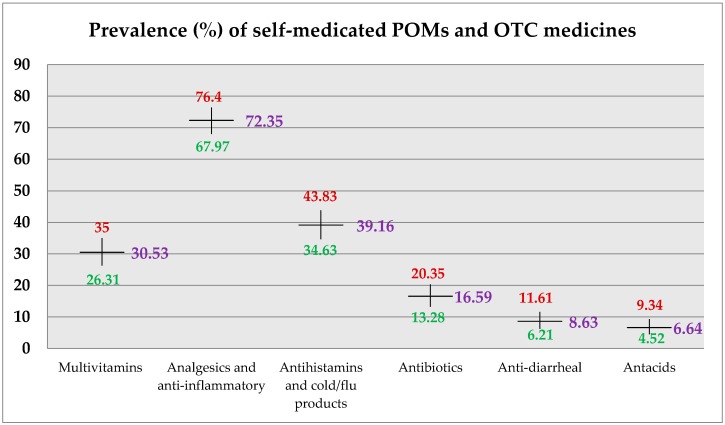
Prevalence of self-medicated POMs and OTC medicines.

The prevalence of multivitamins use was reported at 30.53% (26.31–35% for 95% CI). The term ‘dietary supplement’ included only multivitamins. The prevalence of analgesics use, which included paracetamol and NSAIDs, was reported at 72.35% (67.97–76.4%); antihistamines and cold/flu products was 39.16% (34.63–43.83%). The prevalence of antibiotics and anti-diarrheal use among students was found to be 16.59% (13.28–20.35%) and 8.63% (6.21–11.61%), respectively. For antacids use, the prevalence was documented at 6.64% (4.52–9.34%). The detailed summary of self-medication information is graphically represented in Figure 2 and tabulated in Table 4.

### 3.4. Attitudes Towards Self-Medication Practice

The study documented students’ attitude towards self-medication practice. More than a third of students (N = 158, 35.1%) responded positively to SM practice by mentioning ‘mild problems’ that could be treated by SM. Few students (N = 64, 14.2%) indicated that they had ‘previous experience with such medicines’, hence, they felt more poised to self-medicate. Similarly, those who had a negative attitude towards SM practice believed that consultation with a physician was essential to stay healthy (N = 119, 26.4%). A small segment of students (N = 68, 15.2%) was concerned with ADRs and did not self-medicate. Most students (N = 366, 81.3%) highlighted that they were against SM practice in principle, but agreed that it may be used in rare situations. A small segment of students (N = 57, 12.7%) favored SM practice at all times. The details are tabulated in Table 5.

The association between gender and the source of information regarding SM was statistically significant with the chi square (X^2^) value reported at 22.302 and *p* value less than 0.0001, with a low to moderate effect size, i.e., a phi value reported at 0.223. The association between demographic variable of college and source of information regarding SM was also statistically significant with X^2^ values reported at 13.390 and *p* value less than 0.05, with a weak effect size, i.e., a phi value reported at 0.172. The association of the variable of college was also significant with nature of medications used by students. The value of X^2^ was reported at 6.669 and *p* value was less than 0.05, with a weak effect size, i.e., a phi value reported at 0.122. Moreover, cross-tabulation for the abovementioned three associations had no cell with minimum expected count less than five; therefore, the results are reliable.

Furthermore, cross-tabulation of study year with nature of medications used by students was significant as X^2^ value was reported at 30.421 and *p* value was less than 0.01, with a weak to moderate effect size, i.e., a phi reported at 0.260. Only five cells (27.8%) had a minimum expected count less than five; therefore, the results may be considered reliable. The association between number of siblings and advice regarding SM practice was found to be statistically significant with the X^2^ value reported at 16.079 and *p* value less than 0.05, with a weak effect size, i.e., a phi value reported at 0.189. Only four cells (26.7%) had an expected count less than five; therefore, the results may be considered reliable. The summary of cross-tabulation is presented in Table 6.

## 4. Discussion

This study was conducted among students of pharmacy and medicine colleges at a public sector university in Dammam, Saudi Arabia. The age and marital status of the students represents the characteristic student enrollment in Saudi academia [18,19]. The prevalence of self-medication in both medicine and pharmacy colleges, combined was reported at 26%. This is quite low compared to the prevalence of SM previously reported in allied health students of other public sector universities of Saudi Arabia [18,19]. Furthermore, the prevalence of SM in College of Clinical Pharmacy alone was reported at 19.61%. This figure has been reported for the first time in this population as there is a lack of data pertaining to self-medication practice prevailing among Saudi pharmacy students. A study conducted in pharmacy students of a university in Saudi Arabia reported SM prevalence of 77%, however; the figure is not specific for pharmacy students as it was obtained from students of pharmacy, nursing, and dentistry, combined [20,21]. In regional context, study conducted in the pharmacy college of a university in the UAE and Pakistan reported SM prevalence of 86% and 67.2%, respectively [22,23]. 

The prevalence of SM in College of Medicine was found to be 49.3%. Studies have reported varying prevalence in medicine colleges from 66% to 87% among Saudi universities [18,19,21,23,24,25]. Our study has reported lowest SM prevalence in a medical college of Saudi university as of now. In regional context, the difference in prevalence between pharmacy and medicine colleges within universities can be attributed to the fact that students of medicines are deemed to become future prescribers. Hence, they find themselves more confident in indulging in self-medication. On the other hand, pharmacy students have more knowledge about drugs and their toxicology which may increase reluctance to indulge in the practice [18,25]. Further investigation is warranted.

There was a surge in the use of multivitamins as our study reported a prevalence of 30.53% which is quite high as compared to previously-reported prevalence of 3–5% [18,19,25,26]. In the region, prevalence of multivitamins use in university students of the UAE was reported at 39% [27]. The use of analgesics, such as paracetamol and NSAIDs, was quite high. Our study reported the prevalence at 72.35% which is the second highest figure reported among Saudi universities currently. The prevalence for the same was reported in previous literature in ranges of 28.7% to 80% among other Saudi universities [18,19,22,25]. Our study also reported the prevalence of antibiotics use among students which was documented at 16.59%. This was second to the lowest figure, i.e., 5% previously reported from King Saud University [19]. High prevalence of antibiotic use was previously reported in the range of 30% to 32% from Taibah and Qassim University, respectively [18,21,25]. Additionally, prevalence of antihistamines and cold/flu products use was comparatively higher than figures reported from other universities of the country. Our study reported a prevalence of antihistamines use at 39.16% which was far higher than figures reported from Taibah University (1.1%) and King Saud University (5%) [18,19]. It was second to highest prevalence reported i.e. from Qassim University, (41%) [25]. The prevalence of anti-diarrheals and antacids was reported for the first time from this population.

Self-medication with anti-fungal (N = 1), corticosteroids (N = 2), anti-acne, i.e., Isotretenoin (N = 1), and anti-emetic i.e., ondansetron (N = 2), CNS stimulants i.e., methylphenidate (N = 1) and anti-spasmodic, i.e., mebavarine (N = 1) was also reported. The prevalence of depression and psychoactive stimulants use in pharmacy students of Pakistan was reported at 1.31% [12,28]. Previous studies conducted in Saudi Arabia found a number of students indulged in substance abuse, especially abuse of such products. Studies have reported the growing substance abuse among students of Saudi universities [24,25,29]. In our study, only a single student acknowledged self-medicating with methylphenidate.

The most common source of information regarding SM were pharmacists, as well as physicians. This finding was also statistically associated with gender and colleges. Pharmacists were the preferred choice for males to seek SM-related information. Females were more inclined towards physicians. Pharmacy students sought information regarding SM from pharmacists. Similarly, students from the College of Medicine highlighted their preference of a physician for such information. Since students of medical college were taught by physicians, it may have promoted confidence in seeking such information from physicians [19,20]. The same principle may apply to pharmacy students.

The majority of the students self medicated with OTC medicines, however, a third proportion of the students used both OTC and POMs. This finding was statistically associated with college and year of study. Medical students and those studying in the 2nd Professional year self-medicated with POM, more than their counterparts. One possible explanation regarding this association could be the fact that physicians in this part of the globe have sole prescribing rights and are deemed by society to prescribe medicines. Medical students who were taught by physicians may find themselves in a much more comfortable position to use POMs as compared to pharmacy students since they are ingrained with a prescribing authority. They may be more inclined towards this practice. Students appeared self-medicating with POM in the 2nd year in higher numbers compared to any other study year. The professional education in pharmacy and medicine at the university starts when the students have progressed from the preparatory year into 2nd year. As the students progress in their educational career, they become more educated and informed about the phenomenon. Professional education may influence their perception towards SM practice and use of POMs. As a result, they may develop reluctance in self-medicating with POM progressively. Hence, our study found that the number of students self-medicating with POMs decreased as they progressed in their educational career.

The most common reasons given as a justification to indulge in SM were ‘mild problem’ and ‘previous experience with medicine’. This finding was congruent with previous literature reported from the country, as well as the region [18]. The importance of physician consultation was stated the most common reason against this practice, which was also reported from university students of Pakistan [13]. Contrastingly, an overwhelming majority of students highlighted their stance that they were against SM practice, in principle, but clarified that SM could be used in rare situations. This was a novel finding as previous studies conducted among the same population reported that most of the students of Jazan University (52.6%) and Taibah University (87%) appeared totally against the practice [18,23]. It can be deduced from this finding that students at Imam Abdulrahman Bin Faisal University understand importance of responsible self-medication.

## 5. Conclusions

The prevalence of self-medication in pharmacy students was quite low and has been reported for the first time. The prevalence of self-medication in medical students was the second lowest reported from a Saudi university, currently. Students mostly self-medicated with OTC drugs however, the use of antibiotics was also observed. The prevalence of antibiotics use was the second lowest among Saudi universities, however, and some students reported using corticosteroids and isotretenoin, which are quite dangerous if self-medicated. Students had a positive outlook towards pharmacists as drug information experts. Our study reported an increased use of multivitamins. Full-scale studies are needed to document the prevalence of multivitamins use in this population. The most common reasons to self-medicate were ‘mild problems’ and ‘previous experience with medicine’. Students of medicine and pharmacy colleges at IAU understand importance of responsible self-medication.

## Figures and Tables

**Table 1:** Correlation matrix.

		V1	V2	V3	V4	V5	V6	V7	V8	V9	V10	V11
Correlation	**V1**	1.000	−0.433	−0.056	−0.012	−0.009	−0.071	0.039	0.169	0.203	−0.029	0.130
	**V2**	−0.533	1.000	0.032	0.075	0.124	0.111	−0.042	−0.240	−0.179	−0.014	−0.237
	**V3**	−0.056	0.032	1.000	0.124	0.111	0.073	0.097	−0.028	0.024	0.151	−0.043
	**V4**	−0.012	0.075	0.124	1.000	0.139	0.280	0.022	−0.103	−0.084	−0.032	−0.094
	**V5**	−0.009	0.124	0.111	0.139	1.000	0.207	0.003	−0.110	−0.108	0.062	0.020
	**V6**	−0.071	0.111	0.073	0.280	0.207	1.000	−0.004	−0.121	−0.075	0.121	−0.127
	**V7**	0.039	−0.042	0.097	0.022	0.003	−0.004	1.000	0.017	0.001	0.044	−0.018
	**V8**	0.169	−0.240	−0.028	−0.103	−0.110	−0.121	0.017	1.000	0.445	−0.194	0.308
	**V9**	0.203	−0.179	0.024	−0.084	−0.108	−0.075	0.001	0.445	1.000	0.088	0.188
	**V10**	−0.029	−0.014	0.151	−0.032	0.062	0.121	0.044	−0.194	0.088	1.000	−0.029
	**V11**	0.130	−0.237	−0.043	−0.094	0.020	−0.127	−0.018	0.308	0.188	−0.029	1.000
Sig. (1-tailed)	**V1**		0.000	0.120	0.402	0.424	0.068	0.207	0.000	0.000	0.268	0.003
	**V2**	0.000		0.250	0.055	0.004	0.009	0.186	0.000	0.000	0.387	0.000
	**V3**	0.120	0.250		0.004	0.009	0.060	0.020	0.279	0.302	0.001	0.181
	**V4**	0.402	0.055	0.004		0.002	0.000	0.319	0.015	0.038	0.247	0.023
	**V5**	0.424	0.004	0.009	0.002		0.000	0.472	0.010	0.011	0.093	0.338
	**V6**	0.068	0.009	0.060	0.000	0.000		0.463	0.005	0.057	0.005	0.003
	**V7**	0.207	0.186	0.020	0.319	0.472	0.463		0.356	0.488	0.174	0.354
	**V8**	0.000	0.000	0.279	0.015	0.010	0.005	0.356		0.000	0.000	0.000
	**V9**	0.000	0.000	0.302	0.038	0.011	0.057	0.488	0.000		0.031	0.000
	**V10**	0.268	0.387	0.001	0.247	0.093	0.005	0.174	0.000	0.031		0.269
	**V11**	0.003	0.000	0.181	0.023	0.338	0.003	0.354	0.000	0.000	0.269	
a. Determinant = 0.359												

**Table 2:** Pattern matrix.

Variable	Component				
	1	2	3	4	5
**V1**	0.814	−0.050	−0.289	0.010	0.071
**V2**	0.748	−0.056	0.165	−0.018	0.065
**V3**	0.564	0.018	−0.010	−0.130	−0.174
**V4**	−0.062	0.707	−0.292	−0.059	0.186
**V5**	−0.054	0.706	0.077	0.008	−0.046
**V6**	0.026	0.620	0.103	0.018	−0.125
**V7**	−0.089	−0.003	0.916	−0.083	0.028
**V8**	0.007	0.095	0.011	−0.854	0.022
**V9**	−0.094	0.064	−0.082	0.796	−0.046
**V10**	−0.105	−0.127	−0.069	−0.133	0.854
**V11**	0.189	0.208	0.309	0.186	0.518

**Table 3:** Demographic information.

Demographic Information (N = 450)	Sample (N)	Percentage (%)
**Age**		
Less than 18 years	5	1.1
Between 18 and 23 years	427	94.9
Between 24 and 30 years	18	4
**Gender**		
Male	249	55.3
Female	201	44.7
**College**		
Clinical Pharmacy	153	34
Medicine	297	66
Study year		
**Preparatory year**	8	1.7
2nd year	98	21.8
3rd Year	138	30.7
4th Year	89	19.8
5th Year	103	22.9
6th year	14	3.1
**Marital status**		
Single	385	85.6
Married	61	13.6
Other	4	0.8
**Number of children**		
1 to 2	20	4.4
3 or more	2	0.4
Married without children	43	9.6
I am Single/not applicable	385	85.6
**Resident status**		
Living with family	375	83.3
Living alone (University accommodation)	75	16.7
**Siblings**		
Between 1 and 2 siblings	40	8.9
Between 3 and 5 siblings	195	43.3
Between 6 and 8 siblings	124	27.6
More than 8 siblings	63	14
No sibling	28	6.2
**Illness**		
Diabetes Mellitus (DM)	7	1.6
Hypertension (HTN)	7	1.6
Thyroid disorders	4	0.9
Anemia	7	1.6
Asthma	8	1.8
Glucose 6–phosphate dehydrogenase (G6PD)	10	2.2
Skin Diseases	1	0.2
Depression	6	1.3
No disease (healthy)	400	88.8

**Table 4:** Self-medication information.

Self-Medication Information (N = 450)	Sample (N)	Percentage (%)
**Did you practice self-medication during the last month?**		
Yes	248	55.1
No	202	44.9
**Frequency of practice?**		
No SM	87	19.3
Rarely (Once a month)	290	64.5
Frequently (Once every two week)	54	12
Very frequently (Once a week)	19	4.2
**Source of Information**		
Pharmacist	124	27.6
Physician	106	23.6
Medical professionals	43	9.5
Family and Friends	61	13.6
Internet	116	25.7
**Nature of the medication used**		
Prescription only medicines (POMs)	82	18.2
Non-prescription (OTC) drugs	216	48
Both	152	33.8
**Place of obtaining medications for self use**		
Pharmacy store	306	68
Friends	5	1.1
Available in house	139	30.9
**Symptoms experienced**		
Headache, fever and pain, dysmenorrhea	64	14.2
Cold and Flu Allergy	16	3.6
Gastric symptoms (Diarrhea/constipation/ indigestion)	11	2.4
Throat, RTI and skin infection	9	2
None	57	12.7
More the one	293	65.1
**Types of medications used for SM**		
Analgesics/Antipyretics (Paracetamol/NSAIDs)	102	22.7
Antibiotics (Amoxicillin, Cefexime)	6	1.3
Cough and Flu (Psuedoephedrine)	10	2.3
Anti-diarrheal/Laxatives (Lactulose)	2	0.4
Anti-histamines (Loratadine, Citirizine)	5	1.1
Multivitamins	13	2.9
CNS stimulants (Amphetamines, Methylphenidate)	1	0.2
I do not remember	53	11.8
More than one medication used	258	57.3

**Table 5:** Student attitudes towards self-medication practice.

Attitudes towards Self-Medication (N = 450)	Sample (N)	Percentage (%)
**Reasons in favor of self-medication practice**		
Mild problems	158	35.1
Previous experience	64	14.2
I find SM practice as taking active role in managing my health	23	5.1
Waiting in queues and time issues	25	5.6
Lack of trust on prescribers	9	2
Self-knowledge is enough to self-medicate rationally	14	3.1
Informed by elders/family members and friends	8	1.8
I am not sure	114	25.3
Combination of above mentioned reasons	35	7.8
**Reasons against self-medication practice**		
Consultation with physician is essential	119	26.4
Risk of adverse drug reactions (ADRs)	68	15.1
Practitioner can diagnose an illness	48	10.7
A patient cannot rationalize SM	18	4
Prescribing a medication is the job of a prescriber	18	4
I am not sure	130	28.9
Combination of above mentioned reasons	49	10.9
**Advice to others regarding self-medication**		
I am always in favor of SM practice	57	12.7
I am against SM but it can be used in rare situations	366	81.3
I am always against SM practice	27	6

**Table 6:** Cross-tabulation between demographic and dependent variables.

Demographic Variable	Source of Information					*p* Value
**Gender**	**Pharmacist**	**Physician**	**Medical professionals**	**Family and Friends**	**Internet**	
Male	89 (68.6)	53 (58.7)	16 (23.8)	34 (33.8)	57 (64.2)	*0.0001*
Female	35 (55.4)	53 (47.3)	27 (19.2)	27 (27.2)	59 (51.8)	
**College**	**Pharmacist**	**Physician**	**Medical professionals**	**Family and Friends**	**Internet**	
Clinical Pharmacy	54 (42.2)	23 (36)	12 (14.6)	21 (20.7)	43 (39.4)	*0.01*
Medicine	70 (81.8)	83 (70)	31 (28.4)	40 (40.3)	73 (76.6)	
	**Nature of medications used by students**					
**College**	**(Prescription) Rx drugs**		**(Over-the-counter) OTC drugs**		**Both**	
Clinical Pharmacy	19 (27.9)		84 (73.4)		50 (51.7)	*0.036*
Medicine	63 (54.1)		132 (142.6)		102 (100.3)	
**Study year**	**Rx drugs**		**OTC drugs**		**Both**	
Preparatory year	3 (1.5)		1 (3.8)		4 (2.7)	*0.01*
2nd Year	33 (17.9)		35 (47)		30 (33)	
3rd Year	23 (25.1)		70 (66.2)		45 (46.6)	
4th Year	7 (16.2)		46 (42.7)		36 (30.1)	
5th Year	14 (18.8)		57 (49.4)		32 (34.8)	
6th Year	2 (2.6)		7 (6.7)		5 (4.7)	
	**Advice regarding self-medication practice**					
**Siblings**	**Always in favor**		**Against SM but can be used when necessary**		**Always against the practice**	
Between 1 and 2 siblings	9 (5.1)		28 (32.5)		3 (2.4)	*0.041*
Between 3 and 5 siblings	25 (24.7)		159 (158.6)		11 (11.7)	
Between 6 and 8 siblings	12 (15.7)		109 (100.9)		3 (7.4)	
More than 8 siblings	7 (8)		47 (51.2)		9 (3.8)	
No sibling	4 (3.5)		23 (22.8)		1 (1.7)	
